# Preprocessing of emotional visual information in the human piriform cortex

**DOI:** 10.1038/s41598-017-09295-x

**Published:** 2017-08-23

**Authors:** Patrick Schulze, Anne-Kathrin Bestgen, Robert K. Lech, Lars Kuchinke, Boris Suchan

**Affiliations:** 1 Department of Neuropsychology, Institute of Cognitive Neuroscience, Faculty of Psychology, Ruhr-University Bochum, 44801 Bochum, Germany; 2 Department of Experimental Psychology and Methods, Faculty of Psychology, Ruhr-University Bochum, 44801 Bochum, Germany; 3 Department of Methology and Evaluation, International Psychoanalytic University, 10555 Berlin, Germany

## Abstract

This study examines the processing of visual information by the olfactory system in humans. Recent data point to the processing of visual stimuli by the piriform cortex, a region mainly known as part of the primary olfactory cortex. Moreover, the piriform cortex generates predictive templates of olfactory stimuli to facilitate olfactory processing. This study fills the gap relating to the question whether this region is also capable of preprocessing emotional visual information. To gain insight into the preprocessing and transfer of emotional visual information into olfactory processing, we recorded hemodynamic responses during affective priming using functional magnetic resonance imaging (fMRI). Odors of different valence (pleasant, neutral and unpleasant) were primed by images of emotional facial expressions (happy, neutral and disgust). Our findings are the first to demonstrate that the piriform cortex preprocesses emotional visual information prior to any olfactory stimulation and that the emotional connotation of this preprocessing is subsequently transferred and integrated into an extended olfactory network for olfactory processing.

## Introduction

The interaction between olfactory and visual information contributes to an effective perception of odors. For example, odor identification is improved by additional verbal information^1^ and odor detection is enhanced by the presentation of an odor-source congruent picture (e.g. a picture of oranges, when an orange odor is presented)^2^. Thus, the olfactory system strongly benefits from information provided by the visual system. Furthermore, a recent study using immediate early gene imaging in mice showed context-driven activation of visual information in the piriform cortex as the main region of olfactory perception^3^. This activation was found in the absence of any olfactory stimuli, but the visual information had been associated to the olfactory information in a prior learning phase. Similar results were also found in humans. Olfactory imagery as well as seeing and reading a label of an odor source activates the primary olfactory cortex, again without any physical contact to an odor^4, 5^. These results are an indication for an adapted olfactory system, which processes visual information associated with olfactory stimuli. Another remarkable recently detected characteristic of the olfactory system is the function to generate predictive templates^6^. These templates facilitate the preprocessing and the generating of expectations before incoming olfactory information.

**Figure 1:**
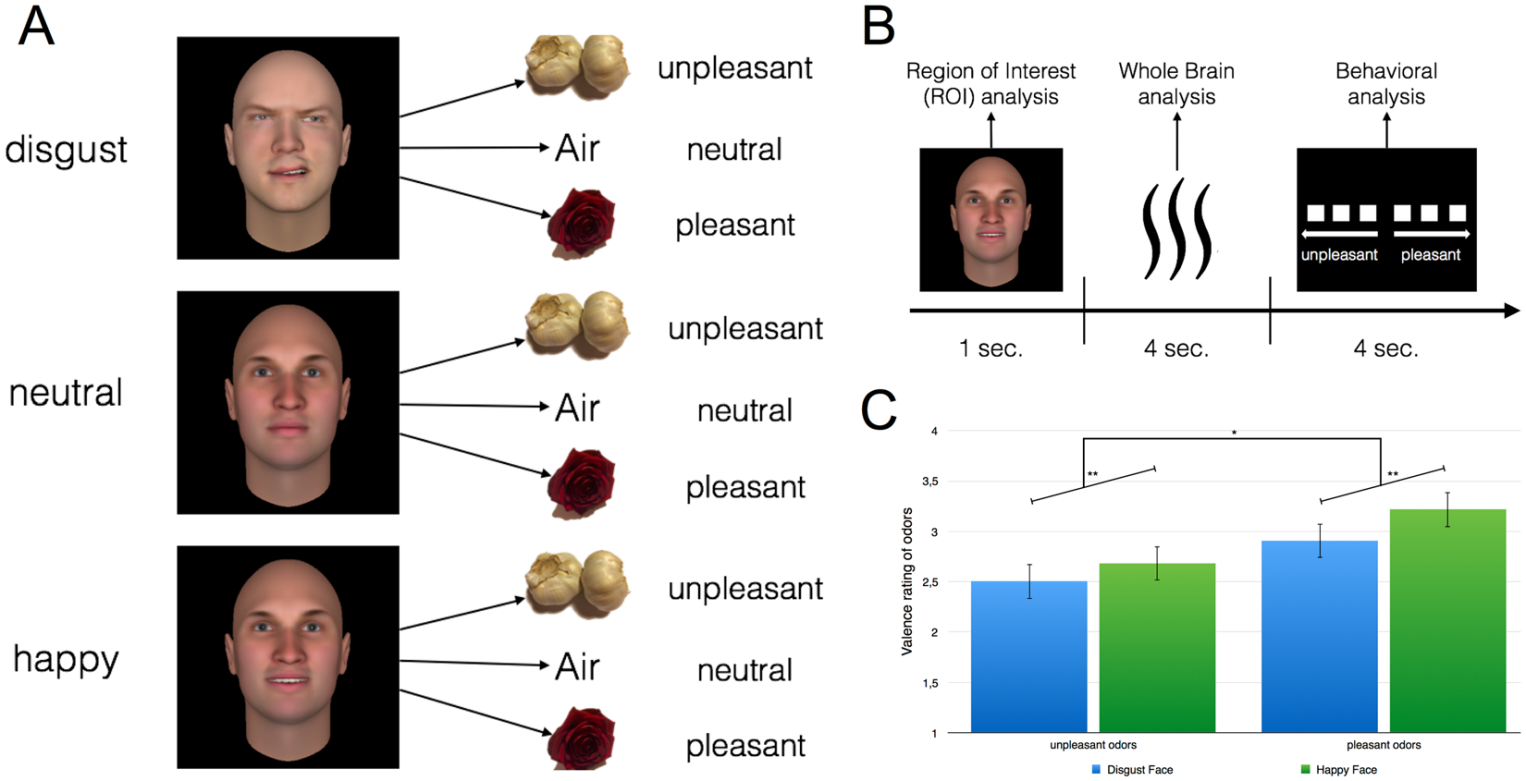
Experimental design and behavioral data. (**A**) Disgusted, neutral and happy facial expressions from the Bochumer Emotional Stimulus Set^10^ were used. Each facial expression was paired with an unpleasant, neutral or pleasant odor. (**B**) For each run, subjects were presented with an emotional facial expression (disgusted, neutral or happy) for 1 seconds, followed by an odor (unpleasant, neutral or pleasant) for 4 seconds, which in the following had to be rated for its valence within 4 seconds. Region of interest analysis was conducted for visual presentation of facial expressions before odor presentation. For odor presentation, a whole brain analysis was conducted. (**C**) Valence ratings revealed a significant main effect of preceding facial expressions on the perceived odor valence. **Signifies a statistically significant difference at the threshold 0.01.

On the basis of these findings in mice and humans, our study directly addresses the question whether emotional visual information, which has not been associated with an odor before, is preprocessed in the human piriform cortex before the actual perception of an odor takes place. Moreover, odor perception per se is strongly influenced by emotional information^7^. For example emotional verbal labels can invert the perceived valence of odors, changing formerly pleasant odors to unpleasant and vice versa^8^. In humans, automatic emotional information processing is able to modulate subsequent processing stages very quickly. Thus, the use of emotional facial expressions as primes or cues for incoming olfactory stimuli should lead to a faster and reliable activation of the piriform cortex. This advantage of emotional stimuli can be further considered as an indicator of preprocessing of visual information by the olfactory system. Additionally, we are interested in the question whether the emotional valence of visual primes is also integrated in the olfactory processing by additional regions of the olfactory network and whether this integration influences the perceived valence of an odor. To examine the preprocessing and integration of emotional visual information into olfactory processing, 12 odors of different emotional valences (unpleasant, neutral (air), pleasant) were presented and primed with emotional facial expressions (disgust, neutral, happy) (see Fig. 1A and B). Healthy human subjects underwent a cross-modal affective priming inside a functional magnetic resonance imaging (fMRI) scanner and the odors were presented with an fMRI compatible olfactometer^9^.

## Materials and Method

### Participants

Eighteen neurologically healthy human subjects participated in the current study. One participant had to be excluded from the analysis because of technical difficulties, resulting in 17 participants that were included in the analysis (8 male and 9 female; mean age: 21.8 years; range: 18–28). All participants were right-handed and had normal or corrected-to-normal vision. All subjects self-reported a normal sense of smell and gave informed written consent after a detailed oral and written explanation of the procedures. The study received ethical approval by the Ethics Board of the Faculty of Psychology of the Ruhr-University Bochum, which conforms to the Declaration of Helsinki.

### Stimuli

#### Emotional visual Stimuli

Face stimuli were chosen from the Bochum Emotional Stimulus set (BESST), which contains high-resolution colored pictures of bodies and faces depicting either a neutral expression or one of the six basic emotions^10^. The faces were created on the basis of pictures of male and female human faces that were laser scanned in 3D using Face Gen Modeller software. For the present study, 54 happy, 54 neutral and 54 disgusted faces (in total 162 faces, 81 female) were selected from the group of 20 year-old faces. Faces were presented in a frontal view, upwards from the neck with no facial and no top hair.

#### Olfactory Stimuli

Olfactory stimuli consisted of six negative and six positive odors from different categories like food, flower and body odors, chosen on the basis of previous work^11^ Ambient air was used as a neutral odor. The negative odors were: Garlic (Garlic oil blend), Sweat (Isovaleric acid), Raw meat (3-Acetyl-2,5-dimethylthiophenene), Onion (Onion oil, artificial), Faeces (Skatole), and Vinegar (Acetic acid) and the positive odors were: Rose (Rose absolute, Maroccan), Lemon (Lemon oil), Caramel (Ethyl maltol), Grass (cis-3-Hexen-1-ol), Mint (L-Carvone, 99%) and Lavender (1-Octen-3-yl acetate). Each odor was prepared as a liquid solution. Odors were presented using a custom-built, MRI-compatible, air diluted, 24-channel olfactometer^9^. With one digital mass flowmeter and two digital pressure sensors, the pressure and flow of the air stream were continuously and digitally controlled. The air-flow was set at 1.5 l/min and added to normal breathing volume. Due to additional custom built pneumatically controlled pinch valves for each channel, placed right before the participant’s nose within the MRI scanner, fast onset times were achieved. The pressure of the pilot air was set at 5.5 bar to ensure a fast closing of the odor air which was set at 1 bar. The odors were presented via a nasal mask into both nostrils. The olfactometer was synchronized and triggered using MATLAB (Version 7.8.0.347) using the PsychoToolBox^12^ and the Psychtoolbox 4 wrapper for MATLAB’ (http://psychwrapper.sourceforge.net/).

#### Procedure

Following the affective priming task, the participants had to perform 162 trials divided into 3 blocks. Each trial began with the presentation of a facial expression for 1000 ms followed by the presentation of an odor for 4000 ms. After the presentation of an odor, a rating scale appeared for 4000 ms and the participant had to rate the valence of the presented odor on a 6-point scale. The responses were recorded pressing a button on a MRI-suitable response system. Each emotional face category was paired with each emotional odor category, resulting in nine conditions (Happy faces were paired with positive, negative and neutral odors; disgust faces were paired with positive, negative and neutral odors and neutral faces were paired with positive, negative and neutral odors) with 54 trials each. Trials were presented in a randomized order. The experiment took place inside of an MRI scanner and was performed using MATLAB (Version 7.8.0.347) combined with the PsychoToolBox^12^ and the Psychtoolbox 4 wrapper for MATLAB’ (http://psychwrapper.sourceforge.net/).

#### Image acquisition

The experiment was performed using a Philips 3 T Achieva MRI scanner with a 32 channel SENSE head coil. A T1 weighted structural scan was acquired for every participant at the start of the experimental session (220 slices, voxel size = 1 × 1 × 1 mm, TE = 3.8 ms, flip angle = 8°). T2* weighted EPIs were acquired during both tasks in an ascending sequence of 30 slices (voxel size = 2 × 2 × 3 mm, TR = 2100 ms, TE = 30 ms). The first five dummy images at the start of each scanning session were discarded to allow for MRI signal stabilization.

#### Data acquisition and analysis

The functional and structural images were pre-processed using the latest release of SPM8 (http://www.fil.ion.ucl.ac.uk/spm/software/spm8). The preprocessing consisted of a slice-time correction, realigning and unwarping, co-registering the EPIs with the structural scan, segmenting the structural scan into grey and white matter, and normalizing the EPIs to MNI space. The unwarping was employed in order to reduce the distortions that can be caused by magnetic field inhomogeneities resulting from movement. All images were re-sliced into 1.5 × 1.5 × 1.5 mm voxels and then smoothed with a Gaussian kernel of 8 mm full-width half-maximum (FWHM). The pre-processed images then were submitted into a first level GLM analysis, where the blood oxygenation level dependency (BOLD) signal was modeled with the canonical hemodynamic response function. A high-pass filter at 128 s was used to remove low frequency drifts.

Twelve regressors were defined on the first level: Three regressors for the priming conditions (disgust, neutral and happy faces) and nine regressors for the combinations of odor and priming conditions (e.g. unpleasant odor preceded by a happy face). First level contrasts were then defined by subtracting “neutral face” responses from “disgust face” responses, “happy face” responses from “disgust face” responses and “neutral face” responses from “happy face” responses for the priming condition. First level contrasts of the olfactory conditions were defined by subtracting the combination of “neutral face and neutral odor (air)” from 1) “disgust face and unpleasant odor”, 2) “disgust face and pleasant odor”, 3) “happy face and unpleasant odor” and 4) “happy face and pleasant odor”. The resulting contrasts were then used for group inference in the second level random effects analysis. For priming condition, region of interest (ROI) analyses within the bilateral primary olfactory cortex (AAL-segmented brain atlas^13, 14^) was conducted on one-sample t-tests comparing 1) “disgust face” to “neutral face”, 2) “disgust face” to “happy face”, 3) “happy face” to “neutral face” responses. For whole-brain analysis of the odor perception a within-subjects full factorial model with two factors was used, including the factors “faces” (disgust vs. happy), and “odors” (negative vs. positive). Additionally, analyses for individual odors including the factor “faces” were conducted, to examine potential effects driven by single odors. For simplicity, only signal changes for the right piriform cortex will be reported.

Significant interaction clusters in the full factorial analyses and responses of the ROI were then used to extract mean signal changes (in percent) with MarsBaR software (http://marsbar.sourceforge.net/). The statistical maps were analyzed using a FWE-corrected threshold of p < 0.05 with a minimum of 10 voxels per cluster.

Behavioral data were analyzed using a repeated measures ANOVA for both valence ratings and reaction time with emotionality of facial expression (disgust vs. happy) and odor valence (unpleasant vs. pleasant) as within-subject factors. To examine potential effects on valence perception driven by single odors, additional analyses for individual odors were conducted.

All statistical analyses of the behavioral data and mean signal changes were performed using SPSS Statistics 22 (IBM Inc.).

## Results

**Figure 2:**
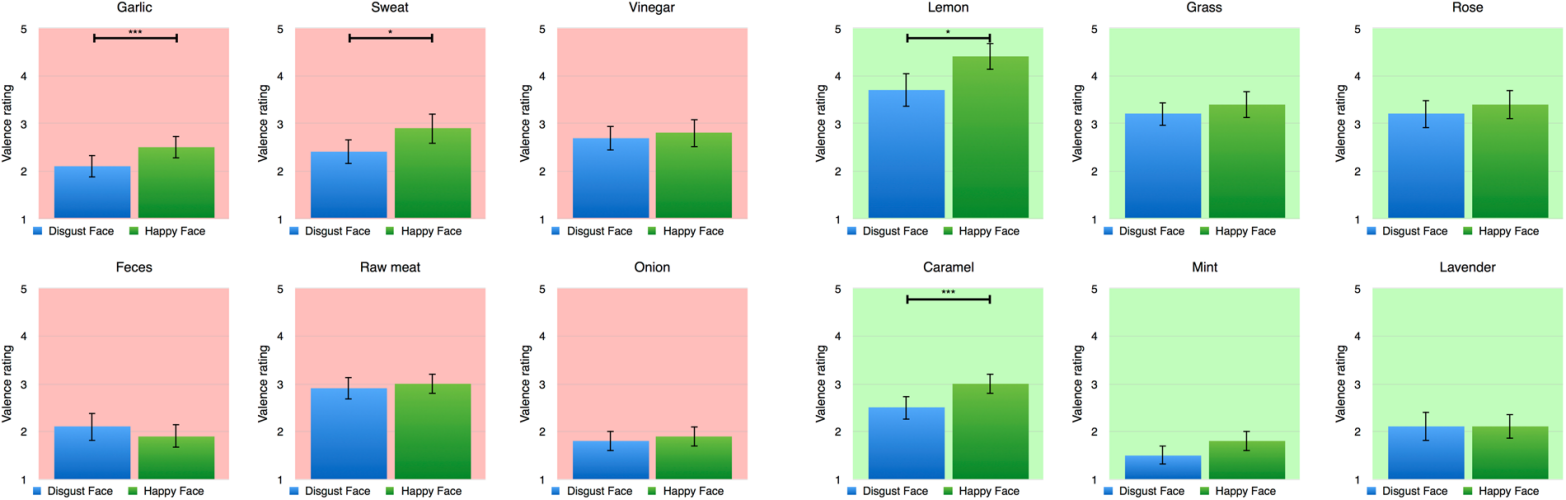
Priming effects on valence perception driven by single odors. All odors but the smell of feces at least show a tendency of an increased pleasantness following a happy face compared to disgust faces. *Signifies a statistically significant difference at the threshold 0.05 and ***signifies a statistically significant difference at the threshold 0.001.

First, we examined whether affective priming by facial expressions modulates the perception of odors by analyzing the valence ratings conducted after the presentation of each odor. As each emotional facial expression will precede an odor, we predict a shift of the perceived odor valence towards the emotion of the preceding facial expression. This effect might be based on preprocessing of the valence of the visual emotional stimuli e.g. in the amygdala which in turns facilitates further processing in the piriform cortex. The behavioral results confirm this prediction. Using a repeated measures ANOVA for valence ratings, with both face emotionality (disgust and happy) and odor valence (unpleasant and pleasant) as within-subject factors, we observed that odors were rated as less pleasant when preceded by a disgusted face (M = 2.7; SE = 0.136) compared to odors preceded by a happy face (M = 3.0; SE = 0.14; F(1, 16) = 13.805; p = 0.002; η^2^ = 0.463) (see Fig. 1C). The analyses of potential effects on valence perception driven by single odors reveal an overall tendency of the described effect (see Fig. 2). A significant increase in valance ratings can be found for garlic (*t*(16) = −3.2; *p* < 0.001), sweat (*t*(16) = −2.0; *p* = 0.034), lemon (*t*(16) = −1.9; *p* = 0.035) and caramel (*t*(16) = −2.7; *p* < 0.001). The smell of feces is the only odor that shows a reverse tendency. As a manipulation check, we also found that unpleasant odors were rated as less pleasant (M = 2.6; SE = 0.179) than pleasant odors (M = 3.1; SE = 0.149; F(1, 16) = 6.686; p = 0.020; η^2^ = 0.295). For reaction time, a main effect for odors can be found (*F*(1, 16) = 5.6; *p* = 0.031; η^2^ = 0.259). We observe a slower response for unpleasant odors (*M* = 1.0; *SE* = 0.13) than for pleasant odors (*M* = 0.9; *SE* = 0.11).

To conclude, behavioral data showed that affective priming has an influence on the perception of odors and that the manipulation of odor valence was effective.

To dissociate the preprocessing of visual information within the piriform cortex from the integration of this information into the extended olfactory network, we conducted two separate fMRI analyses. A ROI analysis for visual presentation of emotional facial expressions before the presentation of the odor and a whole brain analysis at the time of the subsequent odor presentation (see Fig. 1B).

**Figure 3:**
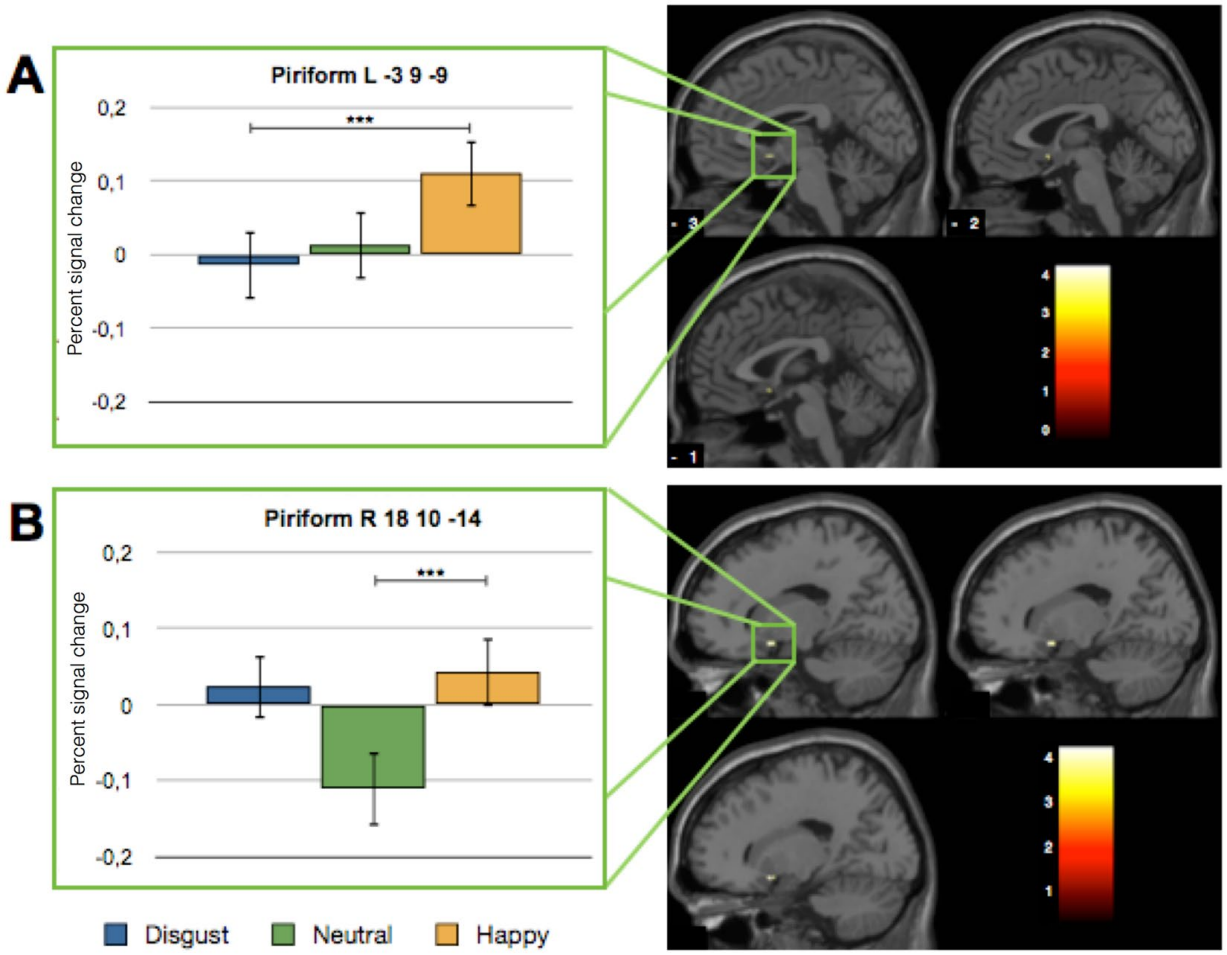
Increased piriform cortex activation prior to odor perception. (**A**) Increased activation for happy faces compared to disgusted faces can be shown within the left piriform Cortex (Piriform L). (**B**) Increased activation for happy faces compared to neutral faces can be shown within the right piriform Cortex (Piriform R). Percent signal change within the small volume corrected ROI (bilateral primary olfactory Cortex) is shown in bar graph: ***signifies a statistically significant difference at the threshold 0.001 corresponding to a FWE-Corrected threshold for small volume of 0.05.

The ROI analysis within the bilateral primary olfactory cortex (POC) using the AAL-segmented brain atlas^13^ was conducted to test the hypothesis whether visual information is processed in the piriform cortex prior to a physical contact with the odor. We found higher activation within the right piriform cortex during the presentation of happy faces compared to neutral faces (t(16) = 4.19; p_FWE-corr._ = 0.038) (see Fig. 3B and Table 1). For a comparison of the activation induced by happy faces and disgust faces, a significant increase within the left piriform cortex was found for happy faces (t(16) = 4.67; p_FWE-corr_ = 0.017) (see Fig. 3A and Table 1). Thus, the present ROI analysis supports our initial hypotheses and reveals that the piriform cortex preprocesses visual information without olfactory stimulation. This might reflect a preparatory process to subsequent odor perception and processing.

**Table 1:** Activation clusters of region of interest (ROI) analysis within the primary olfactory cortex (POC) for visual presentation of facial expressions without olfactory stimulation and activation clusters of the interaction effect from whole brain analysis of subsequent olfactory stimulation dependent on preceding facial expressions.

Contrast	Cluster size	T-score	Cluster-level p-value (FWE-corr.)	Peak-level p-value (FWE-corr.)	MNI coordinates (x, y, z)			Anatomical structure
ROI: Happy Faces vs. Neutral Faces	17	4.19		0.038	18	11	−14	Right piriform Cortex
ROI: Happy Faces vs. Disgust Faces	10	4.67		0.017	−3	9	−9	Left piriform Cortex
Whole Brain: Face Emotionality * Odor Emotionality	448	8.03	<0.001		21	12	−17	Right piriform Cortex
		7.79			18	−3	−15	Right Amygdala
		5.65			27	5	−17	Right Amygdala
	132	6.65	<0.001		−18	−6	−14	Left Hippocampus
	42	6.28	0.001		−38	26	−2	Left tri. inf. frontal Gyrus
	15	5.89	0.007		−26	32	−14	Left inf. OFC
	20	5.81	0.004		−27	5	−15	Left Amygdala
	12	5.70	0.009		−35	23	−17	Left inf. OFC

Cluster size = number of contiguous voxels surviving FWE-correction; FWE-corr. = Family Wise Error corrected for multiple comparisons at threshold of 0.05; MNI coordinates = standard coordinates by the Montreal Neurological Institute; tri. = triangular; OFC = orbitofrontal cortex; inf. = inferior.

**Figure 4:**
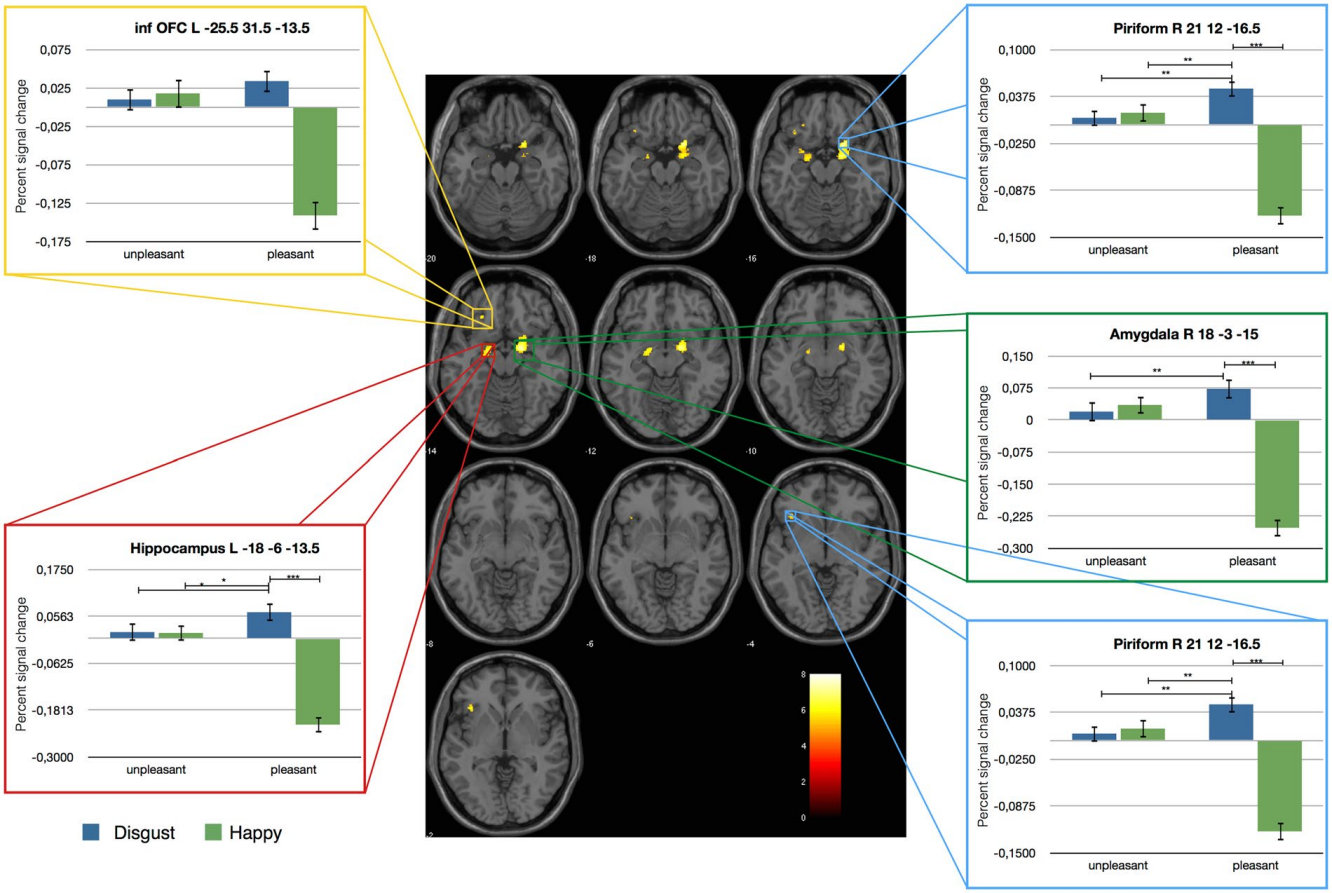
Activation pattern of the whole brain analysis for the olfactory network. The whole brain fMRI analysis revealed a consistent and specific activation pattern throughout the olfactory network and the triangular inferior frontal gyrus, showing that the emotionality of preceding facial expressions has only an impact on the processing of pleasant odors. Percent signal change for the main areas of activation is shown in the bar graphs: left inferior orbitofrontal Cortex (inf OFC L), left hippocampus, left triangular inferior frontal gyrus (tri inf frontal Gyrus L), right piriform Cortex (Piriform R) and right amygdala. ***Signifies a statistically significant difference at the threshold 0.001 corresponding to a FWE-Corrected threshold of 0.05.

**Figure 5:**
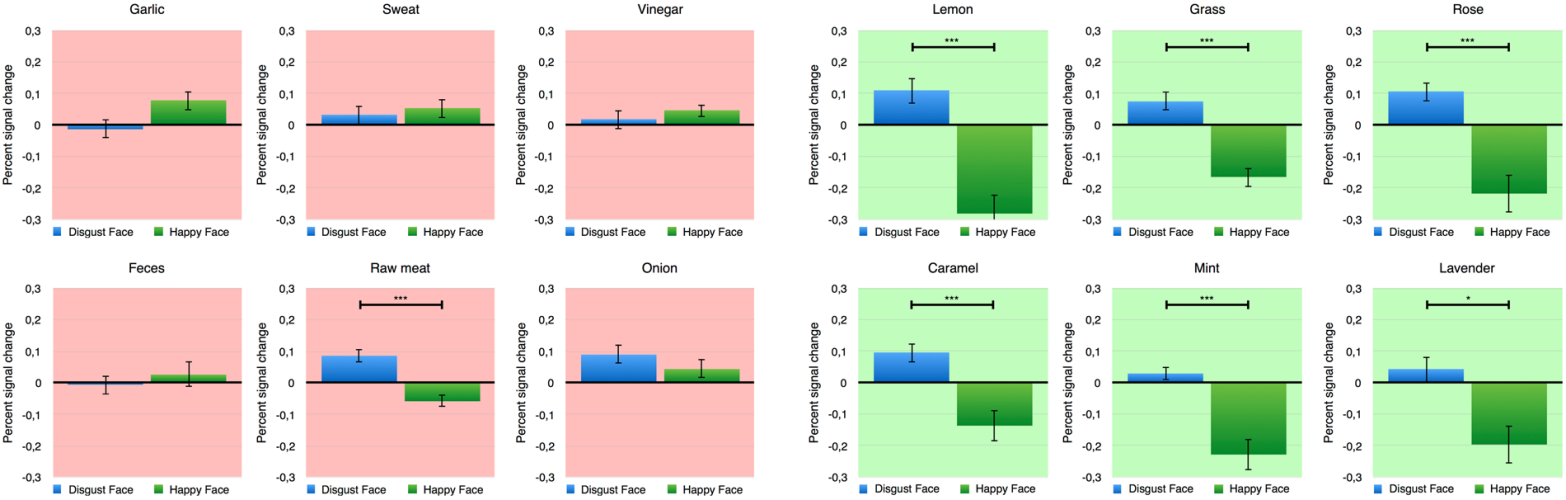
Integration effects of facial expressions to odor perception on activation in the right piriform cortex for single odors. The effect can be found for all pleasant odors but not for unpleasant odors except raw meat. *Signifies a statistically significant difference at the threshold 0.05 and ***signifies a statistically significant difference at the threshold 0.001.

The whole brain fMRI analysis was conducted to determine whether and how the emotional visual information is integrated into odor processing. Here, in contrast to the ROI analysis, we concentrated on the time of the actual odor perception. The whole brain analysis revealed a significant interaction effect between the emotionality of the facial expression and the valence of the odor for the piriform cortex and amygdala of the right hemisphere and amygdala, hippocampus, inferior orbitofrontal cortex and triangular inferior frontal gyrus of the left hemisphere (see Fig. 4 and Table 1). This interaction effect showed an overall consistent pattern of activation. While the processing of unpleasant odors seems to be unaffected by the preceding facial expression, for the processing of pleasant odors, the described regions show increased activation when primed with a disgust face compared with a happy face. In sum, as expected, the whole brain analysis revealed a consistent and specific activation pattern throughout the extended olfactory network. Interestingly, the emotionality of preceding facial expressions only had an impact on the processing of pleasant odors. The consistency of this integration effect is further reassured by the analyses of signal changes for individual odors (see Fig. 5). This effect can be found for all pleasant odors: Lemon (t_(14)_ = 4.4; p < 0.001), grass (t_(14)_ = 4.7; p < 0.001), rose (t_(14)_ = 3.9; p = 0.002), caramel (t_(14)_ = 3.2; p = 0.006), mint (t_(14)_ = 3.9; p = 0.002) and lavender (t_(14)_ = 2.4; p = 0.028). For all unpleasant odors, except raw meat (t_(14)_ = 4.0; p < 0.001), this effect cannot be shown and signal changes do not reach significance. For simplicity, only signal changes within the right piriform cortex are reported for individual odors as this structures showed highest activation if compared to the neutral condition.

## Discussion

The results of this study clearly demonstrate that emotional visual information is preprocessed in the human piriform cortex prior to any physical contact or pairing with an odor. Furthermore, for subsequent olfactory processing, the results show that the emotionality of the faces change the processing of pleasant odors, while unpleasant odors are unaffected. This is of specific interest as the visual stimuli have not been directly associated with any olfactory stimuli. The behavioral data reveal that valence perception of odors is shifted towards the emotional connotation of the facial expression.

As demonstrated by these results, odor processing, especially the perception of valence, is driven by experience and expectation, which includes contextual information^15^. However, up to now, the integration of visual and olfactory information resulting in the modulation of odor processing has mainly been shown for simultaneously presented stimuli. This study effectively fills this gap and gives more insight into the effect of preprocessing, integration and the perceptual value of visual primes on the olfactory system. The behavioral results reveal that the emotion of a facial expression influences the valence rating of an odor. The same odors primed with disgusted faces were perceived as less pleasant than those primed with happy faces. Facial expressions seem to be used as cues to generate an expectation for an incoming olfactory stimulus. There has been no association between the emotional visual input and the olfactory stimulus created before. Indirect processing of these incoming information e.g. via the amygdala might therefore be responsible for these findings. While the amygdala might have pre-processed the incoming emotional information, further processing of this information has been handled by the piriform cortex. Here, a similarity can be drawn to findings from odor imagery. When imagining a pleasant odor, humans tend to take a larger sniff in contrast to a smaller sniff for imagining of unpleasant odors^16, 17^. Thus, seeing a disgusted face will lead to the expectation of being about to smell an unpleasant odor. Similar to imagery, expectations modulate the actual perception and with regard to the different sniffing behavior in the odor imagery study, this probably takes place at an early stage in odor processing. Concerning this, data of the ROI analysis expand the behavioral findings and reveal the key finding of this study, namely the piriform cortex activation for emotional visual information prior to any physical contact with an odor. The observed activation of the left piriform cortex for happy faces is supported by findings from the literature^4, 18, 19^, whereas activations within the right piriform cortex are associated with olfactory memory and familiarity^20, 21^. The observed activation patterns of the piriform cortex also dovetail nicely with results of an animal study that revealed context-evoked neural activation patterns in the piriform cortex in the absence of olfactory stimulation^3^ and expand its function of generating unimodal predictive templates^6^ to a cross-modal preprocessing. Additionally, the whole brain analysis enables us to uncover the regions that are involved in the subsequent integration of emotional visual information in the piriform cortex. Confirming our initial hypotheses, we found activations in the main regions of the olfactory network such as piriform cortex, orbitofrontal cortex, amygdala and hippocampus. Similar regions are found in fMRI studies on odor imagery and olfactory-visual integration^2, 4^. This consistent activation pattern observed for the processing of emotional visual information points to a joint processing in the extended olfactory network. Activation of the piriform cortex without actual olfactory stimulation might be based on preprocessing of emotional visual stimuli in structures like the amygdala which are further integrated in the piriform cortex. Reduction of activation when a happy face preceded a pleasant odor might be explained in terms of an adaptation effect. This effect supports the idea of a preprocessing step e.g. in the amygdala and an adaptation effect when the valence of the olfactory stimuli matches the pre-processed pattern. This finding perfectly matches recent data that show the joint processing of odor valence by a network involving the amygdala, which in conjunction with the piriform cortex, shapes the signal sent to the orbitofrontal cortex^22^. The pattern in the whole brain analysis also demonstrates that the emotion of the facial expression as well as the valence of the odor itself influence odor processing. A substantial basis for this may be the high degree of overlap of neural substrates involved in olfactory and emotional processing^23^ and especially odor valence seems to control the direction of the early stages of olfactory processing^24–26^. The ROI analysis showed an increase in piriform cortex activation in response to happy faces compared to disgusted and neutral faces. This happy face advantage has also been reported in a study using odors as primes for emotional facial expressions^27^. Here, happy faces primed with odors induce higher activation within the default network than neutral and disgusted faces. This reflects findings of a stronger hypoactivation of disgusted faces primed by odors compared to happy faces, within the default network^27^. During the actual odor perception (whole brain analysis), only pleasant odors were influenced by the emotionality of the facial expression, which is in line with findings that unpleasant odors are less vulnerable to an emotional modulation by context information^28^.

## Conclusion

To conclude, our findings support the specific role of the piriform cortex beyond a mere sensory relay up to a higher-order associative brain area. We are the first to demonstrate that the human piriform cortex processes emotional visual information before odor perception. Moreover, we found a common activation pattern in regions of the olfactory network such as piriform cortex, orbitofrontal cortex, amygdala and hippocampus for the subsequent integration of the emotional visual information while processing the actual odor. Matching behavioral results reveal that the emotion of a facial expression influences the valence rating of an odor. Thus, the behavioral observation as well as brain activation data jointly point towards a preprocessed integration of emotional visual information that modulates olfactory processing.
